# Epidemiology and risk factors of *Staphylococcus aureus* CC398 bone and joint infections

**DOI:** 10.1186/s12879-020-05098-0

**Published:** 2020-05-29

**Authors:** Kevin Bouiller, Didier Hocquet, Marlene Sauget, Xavier Bertrand, Catherine Chirouze

**Affiliations:** 1 Maladies infectieuses et tropicales, Centre Hospitalier Régional Universitaire, 25030 Besancon, France; 2 Hygiène hospitalière – Centre Hospitalier Régional Universitaire, 25030 Besançon, France; 3 UMR-CNRS 6249 Chrono-environnement, Université Bourgogne Franche-Comté, 25000 Besançon, France; 4 Centre de Ressources Biologiques Filière Microbiologique de Besançon, Centre Hospitalier Régional Universitaire, Besançon, France

**Keywords:** Prosthetic joint infection, Bone joint infection, Diabetic foot infection, Human ST398, Methicillin susceptible *Staphylococcus aureus*, CC398

## Abstract

**Background:**

A particular ability of the *Staphylococcus aureus* clonal complex 398 (CC398) to cause bone and joint infections (BJI) remains questionable, since some studies have described high prevalence of MSSA CC398 in prosthetic joint infection (PJI) and diabetic foot ostemolyelitis (DFO). Here, we described the long-term epidemiology of CC398 among *S. aureus* isolated from BJI and identified risk factors associated with CC398.

**Methods:**

We included all bone and joint samples with *S. aureus*-positive culture in our university hospital between January 2010 and December 2017. Logistic regression was used for univariate and multivariate analysis.

**Results:**

We identified 124 CC398 isolates among the 958 BJI-associated *S. aureus*. The proportion of CC398 among *S. aureus* increased steadily from 4% in 2010 to 26% in 2017. Only 4 isolates of CC398 were resistant to methicillin. The distribution of BJI types due to CC398 and non CC398 isolates was similar. In multivariate analysis, age (*p* = 0.034, OR = 3.9), McCabe score (*p* = 0.005, OR = 5) and inoculation mechanism (*p* = 0.020, OR = 3.7) were associated with PJI-related CC398. The year of infection (*p* < 0.001, OR = 1.6), Charlson’s score (*p* = 0.001, OR = 1.5) and grade 4 (severe) of the International Working Group of the Diabetic Foot classification (*p* < 0.001, OR = 8.5) were associated with DFO-related CC398.

**Conclusion:**

We highlighted here the emergence and spread of CC398-MSSA in BJI. Patients with comorbidities are at high risk of CC398 MSSA PJI and DFO. The spread of CC398 in the community and hospital settings remains unclear and further epidemiological studies are needed to identify the determinants of its success.

## Background

Bone and joint infections (BJI) are a heterogeneous disease in their pathophysiology, clinical presentation, and management [1] and *Staphylococcus aureus* is the most common pathogen in almost all types of BJIs.

Foot ulcers are common in diabetic patients and bone infection is a major causal factor for lower-limb amputation [2]. Prosthetic joint infection (PJI) remains a dreaded complication following total joint arthroplasty. PJI is reported in approximately 0.3–1.9% of all total arthroplasties.

In recent years, infections with *S. aureus* clonal complex 398 (CC398) have emerged worldwide. Whereas Methicillin-Resistant *S. aureus* (MRSA) CC398 is associated with livestock and most often responsible of colonization and mild infection in humans and animals [3], Methicillin-Susceptible *S. aureus* (MSSA) CC398 is a frequent source of infections in humans, and was described frequently in severe infections such as bloodstream infections (BSI) [4, 5]. Specifically, the incidence of BSIs due to MSSA CC398 has been increasing since 2007 in France [6].

The frequency of CC398 PJI isolates varied from 1.8 to 14% [7, 8] whereas 21.7% of isolates belonged to CC398 in patients with DFO [9]. Since several studies have documented a high prevalence of MSSA CC398 in prosthetic joint infection (PJI) and in diabetic foot osteomyelitis (DFO) [6, 7], a particular ability of this clone to cause BJIs remains questionable. However, risk factors for ST398 BJI remain largely unknown. In the study of Valour et al., no difference was observed between patients with MSSA ST398 and non ST398 PJI, excepted for outcome with a less treatment failure in patients with MSSA ST398 infection.

In this study, the prevalence of CC398 among BJI-related *S. aureus* was determined and patients with BJI-related *S. aureus* CC398 were compared to patients with BJI-related *S. aureus* non-CC398, in a university hospital over 8 years, to identify risk factors associated to infections with CC398. A focus was made on patients with PJI and DFO.

## Methods

### Inclusion

All bone and joint bacteriological samples (per-cutaneous joint fluid aspiration, bone or joint surgical sample) with *S. aureus* positive culture between 1st January 2010 and 31 December 2017 in a French university hospital were retrospectively included.

### Definitions

BJI diagnosis was based on clinical and biological evidence of infection. We extracted the clinical information from the electronic medical records of the patients.

BJIs were classified as follow: (i) orthopaedic implant (i.e. PJI or peripheral or vertebral osteosynthesis) (ii) DFO (iii) BJI related to decubitus ulcer (iv) BJI related to peripheral vascular disease (v) osteomyelitis and (vi) arthritis.

Acute (≤ 4 weeks) and chronic infections were defined on the basis of the duration of signs of infection at the prosthesis site to diagnosis.

The Charlson’s comorbidity index was calculated as previously described [10]. The McCabe score was also used to evaluate underlying illness severity. Patients were classified as rapidly fatal (< 1 year), ultimately fatal (1–4 years) and non-fatal (> 5 years) underlying disease [11].

For DFO, we used the IWGDF/IDSA (International Working Group on the Diabetic Foot/ Infectious Diseases Society of America) classification which defined four grades of severity [12]. Grade 1 and 2 infections were only skin and soft tissue infections (SSTI) and were not included in the current study. Grade 3 defined infections in stable patients with local complication such as joint or bone infection. Grade 4 defined infections in patients with systemic toxicity or metabolic instability.

### CC398 *S. aureus* identification

Only the first isolate from each patient was included. *S. aureus* isolates retrieved from bone and joint cultures were stored at the Centre de Ressources Biologiques Filière Microbiologique, Besançon (CRB-FMB, Biobanque BB-0033-00090). MALDI-TOF MS Microflex LT identified all the isolates as *S. aureus* with a log score value ≥2 according to the manufacturer’s recommendations (Bruker Daltonik GmbH, Bremen, Germany). Cefoxitin susceptibility was determined using the disk diffusion method according to EUCAST recommendations (www.eucast.org). We used a CC398-lineage specific MALDI-TOF MS method to screen all *S. aureus* isolates, as described previously [13, 14] and CC398 status was confirmed by a CC398-lineage specific PCR [15].

### Statistical analysis

All variables were examined by univariate analysis using the chi-square or Fisher’s exact test, as appropriate. Continuous variables were analyzed by Student’s t-test. All statistical tests were two tailed and *p* < 0.05 was considered statistically significant. Multivariate analysis was performed by logistic regression. A backward stepwise selection with an entry and stay level of *p* = 0.2 was used to build the final multivariate logistic regression model. Statistical analyses were computed by SPSS 22.0 (IBM, Armonk, NY, USA).

## Results

**Fig. 1:**
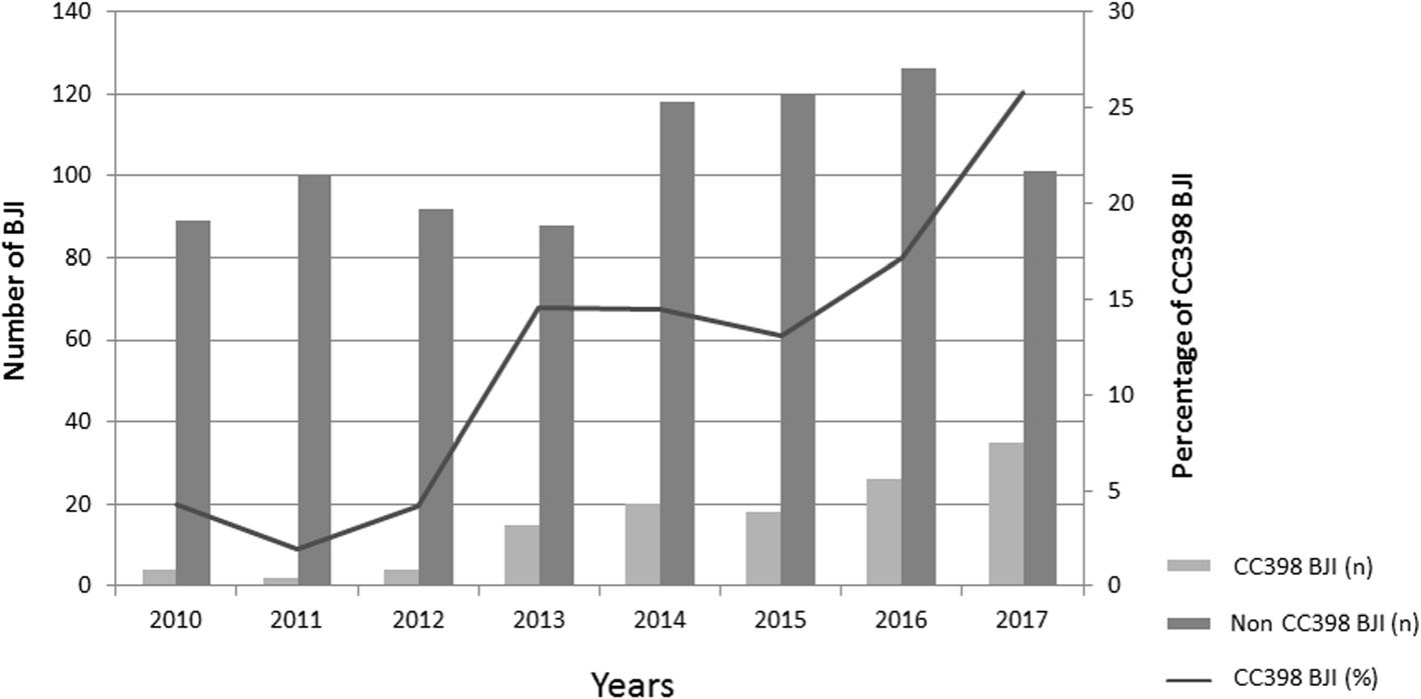
Proportion of *Staphylococcus aureus* (SA) belonging to the complex clonal 398 (*n* = 124) among the SA retrieved in Bone joint and infection (*n* = 958) between 2010 and 2017 in the University Hospital of Besancon, France

Over the eight-year survey, 1080 non-duplicate *S.aureus* BJI were isolated from inpatients of our hospital. Among these, 958 (88.7%) were available for further analysis. Within this *S. aureus* BJI collection, 124 (12.9%) isolates belonged to CC398. Only 4 isolates were resistant to methicillin (one isolate in 2013 and 2015, and 2 isolates in 2017). The proportion of CC398 among *S. aureus* isolates increased steadily from 4% in 2010 to 26% in 2017 (*p* < 0.001) (Fig. 1).

Distribution of the 124 BJI-related *S. aureus* CC398 was showed in Table 1. The distribution of the types of BJIs due to isolates of *S. aureus* CC398 and non-CC398 was similar (Table 1).

**Table 1:** Characteristics of patients with CC398 Bone and joint infections

	CC 398 (*n* = 124)	Non CC 398 (*n* = 834)	Univariate *P* value
Age (years), mean ± SD	62.2 (±18.6)	64.3 (±18.7)	0.24
Male sex, n (%)	91 (73)	573 (69)	0.34
Mc Cabe Score			
Non fatal, n (%)	110 (88.7)	761 (91.2)	0.19
Ultimately fatal, n (%)	13 (10.5)	63 (7.6)	
Rapidly fatal, n (%)	1 (0.8)	10 (1.2)	
MSSA, n (%)	120 (97)	701 (84)	< 0.001
Charlson’s comorbidity score, mean ± SD	2.46 (±2.22)	2.08 (±1.94)	0.073
Year of infection			
2010	4 (3.2)	89 (11)	< 0.001
2011	2 (1.6)	100 (12)	
2012	4 (3.2)	92 (11)	
2013	15 (12)	88 (11)	
2014	20 (16)	118 (14)	
2015	18 (15)	120 (14)	
2016	26 (21)	126 (15)	
2017	35 (28)	101 (12)	
Recent hospitalisation (< 1 yr), n (%)	62 (50)	474 (57)	0.18
Surgery (< 1 yr), n (%)	68 (55)	428 (51)	0.53
BJI Type			
Arthritis, n (%)	8 (6.5)	47 (5.6)	0.87
Osteomyelitis, n (%)	15 (12,1)	119 (14.3)	0.52
Vertebral osteomyelitis, n (%)	0	6 (0.7)	1
Diabetic foot osteomyelitis, n (%)	37 (29.8)	207 (24.8)	0.28
BJI related to decubitus ulcer, n (%)	4 (3.2)	40 (4.8)	0.58
BJI related to peripheral vascular disease	7 (5.7)	101 (12.1)	0.05
Orthopaedic device infection, n (%)	53 (42.7)	314 (37.7)	0.31
including PJI, n (%)	23 (18.6)	121 (14.5)	0.3
including osteosynthesis infection, n (%)	29 (23.4)	184 (22.1)	0.83
including vertebral ODI, n (%)	1 (0.8)	9 (1.1)	1
BJI characteristics			
Evolution delay (months)	28 (±62.9)	37.6 (±101.7)	0.32
Chronic BJI (i.e. evolution delay > 4 weeks), n (%)	49 (75)	270 (68)	0.29
Bacteremiae, n (%)	20 (16)	87 (10)	0.084
BJI mechanisms			
Haematogenous, n (%)	4 (3.2)	49 (5.9)	0.32
Inoculation, n (%)	62 (50)	369 (44)	0.27
Contiguity, n (%)	58 (47)	416 (50)	0.58

A total of 144 patients had a *S. aureus* PJI (12.9%)*,* with 23 due to CC398 isolates (Table 2). Patients with *S. aureus* CC398 PJI were younger (*p* = 0.041), had more severe illness (McCabe score) (*p* = 0.016), were more likely to have early PJI (< 3 months after primary arthroplasty) (*p* = 0.023) and had an inoculation mechanism more frequent (*p* = 0.032) than patients with *S. aureus* non-CC398 PJI. In multivariate analysis, age (*p* = 0.034, OR = 3.9 (1.3–12.0)), McCabe score (*p* = 0.005, OR = 5 (1.6–15.3)) and inoculation mechanism (*p* = 0.020, OR = 3.7 (1.2–11.3)) were associated with PJI due to *S. aureus* CC398.

**Table 2:** Characteristics of patients with CC398 PJI

	CC 398 PJI (*n* = 23)	Non CC 398 PJI (*n* = 121)	Univariate	Multivariate	
			*P* value	*P* value	OR (IC 95%)
**Age (years), mean ± SD**	67.7 (±12.3)	73.6 (±12.3)	**0.041**	**0.034**	**0.9 (0.9–0.99)**
Male sex, n (%)	11 (48)	65 (54)	0.77		
Mc Cabe Score					
Non fatal, n (%)	18 (78)	114 (94.2)	**0.016**	**0.005**	**5.1 (1.6–15.3)**
Ultimately fatal, n (%)	5 (22)	6 (5)			
Rapidly fatal, n (%)	0 (0)	1 (0.8)			
MSSA, n (%)	23 (100)	108 (89)	0.13		
Charlson’s comorbidity score, mean ± SD	2.04 (±1.89)	1.56 (±1.61)	0.27		
Year of infection					
2010	2 (8.7)	14 (11)	0.1	NT	
2011	2 (8.7)	9 (7.3)			
2012	2 (8.7)	18 (15)			
2013	4 (17)	13 (11)			
2014	1 (4.3)	17 (14)			
2015	2 (8.7)	16 (13)			
2016	2 (8.7)	24 (20)			
2017	8 (35)	12 (9.8)			
Recent hospitalisation (< 1 yr), n (%)	13 (57)	74 (60)	0.92		
BJI Type					
Shoulder, n (%)	1 (4.3)	5 (4.1)	1		
Hip, n (%)	16 (70)	75 (62)	0.65		
Knee, n (%)	6 (26)	40 (33)	0.68		
Ankle, n (%)	0	1 (0.8)	1		
Chronic BJI (i.e. evolution delay > 4 weeks)	5 (28)	51 (42)	0.37		
Occurrence of infection after primary arthroplasty					
**Delay (months)**	23 (±49.3)	44.4 (±59.2)	**0.023**	**NT**	
< 3 months, n (%)	14 (61)	44 (36)	0.049	NT	
3–12 months, n (%)	3 (13)	17 (14)	1		
12 months, n (%)	7 (30)	60 (50)	0.14		
Bacteremia, n (%)	8 (35)	32 (26)	0.57		
BJI mechanisms					
haematogenous, n (%)	3 (13)	37 (31)	0.14		
inoculation, n (%)	20 (87)	74 (61)	0.032	0.020	3.7 (1.2–11.3)
Contiguity, n (%)	0	10 (8.3)	0.36		
Clinical features					
Fever, n (%)	10 (43)	48 (40)	0.66		
Sinus tract, n (%)	7 (30)	48 (40)	1		
Abscess, n (%)	3 (13)	12 (9.9)	0.4		
Biological features					
Maximum CRP value (mg/L)	163 (±69.1)	171 (±120)	0.9		

*MSSA* Methicillin susceptible *Staphylococcus aureus, BJI* Bone and joint infection, *NT* Not included in the final model

Of the 244 patients with DFO (25.5%), 37 were infected with CC398 isolates (Table 3). A higher Charlson’s comorbidity score (*p* < 0.001), and grade 4 of the IWGDF/IDSA classification were more frequent in patients with DFO due to *S. aureus* CC398 than in patients with non-CC398 isolates (*p* < 0.001). Multivariate analysis associated the year of infection (*p* < 0.001, OR = 1.6 (1.3–2)), the Charlson’s score (*p* = 0.001, OR = 1.5 (1.2–2)) and grade 4 of the IWGDF/IDSA classification (*p* < 0.001, OR = 8.5 (3.5–20.7)) with DFO due to *S. aureus* CC398.

**Table 3:** Characteristics of patients with CC398 diabetic foot infection

	CC 398 DFI (*n* = 37)	Non CC 398 DFI (*n* = 207)	Univariate	Multivariate	
			*P* value	*P* value	OR (IC 95%)
Age (years), mean ± SD	69.3 (±12.5)	70.3 (±11.3)	0.65		
Male sex, n (%)	28 (76)	166 (80)	0.68		
Mc Cabe Score					
Non fatal, n (%)	34 (91.9)	192 (92.8)	0.81		
Ultimately fatal, n (%)	3 (8.1)	14 (6.7)			
Rapidly fatal, n (%)	0 (0)	1 (0.5)			
MSSA, n (%)	36 (97.3)	171 (83)	0.013	NT	
Charlson’s comorbidity score, mean ± SD	4.51 (±1.71)	3.48 (±1.48)	**< 0.001**	**0.001**	**1.5 (1.2–2)**
Year of infection			**< 0.001**	**< 0.001**	**1.6 (1.3–2)**
2010	0 (0)	29 (14)			
2011	0 (0)	38 (18)			
2012	2 (5.4)	23 (11)			
2013	4 (11)	25 (12)			
2014	6 (16)	24 (12)			
2015	4 (11)	24 (12)			
2016	10 (27)	22 (11)			
2017	11 (30)	22 (11)			
Recent hospitalisation (< 1 yr), n (%)	21 (57)	102 (49)	0.51		
Bacteremia, n (%)	3 (8.1)	12 (5.8)	0.71		
IWGDF-IDSA grade					
3 (moderate), n (%)	14 (38)	174 (84)	**< 0.001**	**< 0.001**	**8.5 (3.5–20.7)**
4 (severe), n (%)	23 (62)	33 (16)			

*MSSA* Methicillin susceptible *Staphylococcus aureus*, *IDSA* Infectious Diseases Society of America, *IWGDF* International Working Group of the Diabetic Foot, *NT* Not included in the final model

## Discussion

In our hospital, the number of BJIs due to *S. aureus* was stable between 2010 and 2013 and then increased in 2014 to stabilize until 2017. However, the prevalence of CC398 among *S. aureus* responsible for BJIs increased steadily from 4% in 2010 to 26% in 2017. Our results describe the increasing trend of MSSA CC398 in BJIs over a 8-year period, echoing what we observed in bloodstream infections in our hospital [4].

The spread of this clone has also been reported in BJIs in other French hospitals with a prevalence varying from 1.8 to 21.7% [7–9, 16, 17]. These differences were explained by different study periods and characteristics of some types of BJI. Indeed, the frequency of CC398 PJI isolates varied from 1.8% from 14% [7, 8], whereas 21.7% of isolates belonged to CC398 in patients with DFO [9]. Interestingly, Senneville et al. showed that *S. aureus* CC398 isolates were significantly more frequent in osteomyelitis than in SSTI for patients with diabetic foot infection and suggested the possible tropism for bone of this clone [9].

This clonal group has been also described in BJIs elsewhere over the world. Uhlemann et al. identified 3 out of 64 (4.7%) CC398 positive samples with osteomyelitis in the United States [18].

Recently, ST398 was found to be the most prevalent clone in BSI-related MSSA in China (14.1%, 32/227), and the prevalence of MSSA ST398 increased from 2013 to 2019 (5.5–18.4%). However, characteristics of BSI including the presence of BJI were not described [5].

Although this clone spread globally, BJI infections with MSSA CC398 had specially been reported in France. It can be explained by the limited surveillance of MSSA in other countries and because most previous international staphylococcal clonal distribution studies have mainly focused on MRSA.

As expected, only 4 isolates were resistant to methicillin. It has been shown that MRSA ST398 and MSSA ST398 belonged to distinct lineages [19]. MRSA ST398 lineage was mainly associated with livestock. It has become a worldwide threat within the past decade and was most often responsible for mild infections, such as SSTI. Few studies had described MRSA CC398 in BJI [20–22]. In contrast, MSSA CC398 was a frequent source of *S. aureus* human infections, and was described frequently in severe infections such as BSI [5, 6, 18, 23]. No contact with livestock was found for the 4 patients with MRSA CC398 isolates. We assume that these 4 isolates were MSSA CC398 from human origin that acquired *mecA* resistance gene but further studies are needed to clarify this point.

The low resistance profile of this clone was confirmed from different studies, who found no particular resistance gene (except for an isolated resistance to macrolides related to the *ermT* gene). Regarding the virulence profile, none of the major, most well-known staphylococcal virulence genes were described in CC398 isolates, except in China, where PVL toxin was found in 80% of CC398 MSSA [5]. However, almost all isolates harboured mobile genetic element belonging to the immune evasion cluster (*chp* and *scn* genes) and the φ3-prophage specific from human lineage and lost in the animal-adapted MRSA CC398 [8, 24, 25].

MSSA CC398 isolates could be more virulent than non-CC398, as suggested by the association of grade 4 of the IWGDF/IDSA classification in patients with *S. aureus* CC398 DFO. However, this may also reflect underlying host comorbidities and immunosuppression. This hypothesis was confirmed in our study with a high Charlson’s score index in patients with SA CC398 DFO, and a high McCabe score in patients with SA CC398 PJI. Similarly, we reported in another study that the 30-day all-cause mortality and McCabe score were higher for patients with MSSA CC398 BSI than those with non-CC398 BSI [23]. Moreover, in a study comparing eight PJIs with MSSA CC398 to 67 PJIs with other clones of MSSA, no demographic and clinical differences were observed between these 2 groups. Conversely, MSSA CC398 BJIs were significantly associated with a lower biological inflammatory syndrome and lower treatment failure rates (0% vs. 37.3%). However a very small number of patients were included and a large number of statistical tests have been performed, which make these results difficult to interpret [8].

The mechanism of acquisition of this clone may be discussed. CC398 MSSA PJI was associated with inoculation mechanisms (mostly post-surgical). Moreover, in our study, more than 90% of patients with DFO were hospitalized in the same medical unit (endocrinology ward) and similarly, patients with PJI or device-associated infection were hospitalized in the same surgical ward. However, other patients (*n* = 40/124) were hospitalized in various wards of our hospital (medical wards *n* = 10, other surgical wards *n* = 25, intensive care units *n* = 5), with no overlapping hospital stays and were taken care by different surgeons in different surgical rooms. Because of the many cases in the same ward and the association with inoculation mechanism after surgery, we cannot rule out hospital cross-transmission. However, the diffusion of this clone in different hospitals in France and in other countries may suggest that this clone spread in both the community and hospital settings. Interestingly, heterogeneity in geographical distribution was observed in France with a prevalence of 3.1 to 23.5% according to the hospital [8]. This heterogeneity could be explained by a greater endemic diffusion in some areas, possibly related to specific routes of transmission.

Our study has some limitations. Firstly, the retrospective collection of data may have introduced information bias. Secondly, we did not determine the population structure of non-CC398, and the control group very probably consisted of genetically diverse *S. aureus*.

## Conclusion

Our local data confirm the emergence and the dissemination of MSSA CC398 in BJI, suggesting a well-adapted fitness of this clone to humans and bone.

Patients with comorbidities are at high risk of MSSA CC398 PJIs and DFOs. The mode of diffusion of this clone in community and hospital remain unclear and further epidemiological studies, based on genome exploration, are needed.

## Data Availability

The datasets used and/or analysed during the current study are available from the corresponding author on reasonable request.

## Abbreviations

BJI: Bone and joint infection
BSI: Bloodstream infection
CC398: Clonal complex 398
DFO: Diabetic foot ostemolyelitis
MRSA: Methicillin-Resistant *S. aureus*
MSSA: Methicillin-Susceptible *S. aureus*
PJI: Prosthetic joint infection
SSTI: Skin and soft tissue infections
